# A Mobile Application for Keyword Search in Real-World Scenes

**DOI:** 10.1109/JTEHM.2019.2935451

**Published:** 2019-08-28

**Authors:** Shrinivas Pundlik, Anikait Singh, Gautam Baghel, Vilte Baliutaviciute, Gang Luo

**Affiliations:** Schepens Eye Research Institute of Mass Eye & EarBostonMA02114USA

**Keywords:** Low-vision aid, mobile application, optical character recognition (OCR), timed instrumental activities of daily living (TIADL) tasks

## Abstract

Keyword search in a cluttered environment is difficult in general, and even more challenging for people with low vision. While magnification can help in reading for low vision people, it does not facilitate efficient visual search due to the constriction of the field of view. The motivating observation for this study is that, in a large number of visual search tasks, people know what are they looking for (i.e., they know the keywords), they just do not know where to find them in the scene. We have developed a mobile application that allows the users to input keywords (by voice or by typing), uses an optical character recognition (OCR) engine to search for the provided keyword in the scene captured by the smartphone camera, and zooms in on the instances of the keyword detected in the captured images, to facilitate efficient information acquisition. In this paper we describe the development and evaluation of various aspects of the application, including comparing the various mainstream OCR engines that power the app, and an evaluation study comparing the app to the conventional optical magnifier vision aid. Normally sighted adults, while wearing blur glasses to lower their visual acuity, performed keyword searches for a series of items ranging from easy to difficult with the app and with a handheld magnifier. While there was no difference in the search times between the two methods for the easier tasks, the app was significantly faster than the magnifier for the difficult tasks.

## Graphical abstract

We present a novel vision assistive mobile app for keyword search in real-world scenes for people with low vision. Users can input search keyword, and the app can localize the keywords in the scene and zoom-in on them to enable faster information acquisition.

## I. Introduction

Visual search is an important, frequently performed visual task in daily life, such as looking for a street name when walking, or finding the calorie content of a food product. Performing visual search in a cluttered environment can be very demanding, even for those with normal vision. [1], [2] It is even more challenging for people with visual impairments [3]–[7] Among the various aspects of daily life that are negatively affected due to visual impairment, the limitations in performing visual search are one of the key challenges that hampers efficient information acquisition. [8], [9] As vision impairment can run the gamut from moderate loss of visual acuity (moderate low-vision) to complete blindness (no light perception), the challenges faced in performing daily tasks vary greatly between patients with different conditions, and between individuals. [10], [11]

**FIGURE 1.**
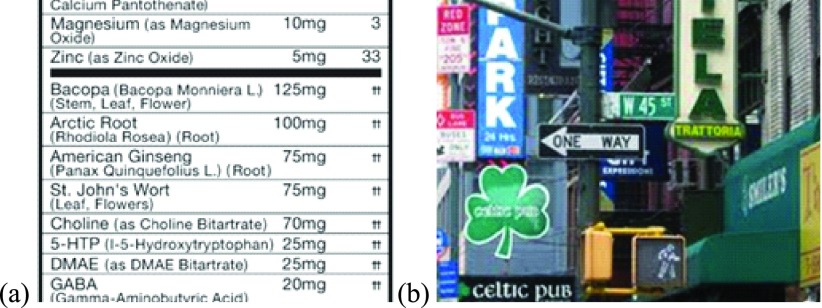
Different aspects of visual search applied to different tasks. (a) Rather than reading through the whole fact sheet, a person often needs to check just a few details about a product, e.g. GABA in this cognitive supplement. (b) In many navigation situations, visually impaired travelers have difficulty knowing where to look to find the needed information amongst all the available options. When using magnification to read distant text, the reduced field of view makes finding the target more difficult in cluttered environments.

Visual search can be considered a spot reading task, which is the act of quickly locating and acquiring a specific piece of information from a scene. People with moderate to severe vision loss (up to the level of legal blindness), such as those who have central vision loss, often rely on magnification for reading or discerning the details of their surroundings. [12], [13] However, increased magnification leads to a smaller field of view, which makes searching for a particular detail within the scene highly inefficient. For example, when searching for a particular ingredient on a product label, the information of interest is only a small portion of the label (Figure 1a). However, one usually needs to go through the list from top to bottom and end-to-end. When using magnification, as is usual for many visually impaired people, this kind of search may take even longer time due to the reduction of the field of view caused by magnification. On the other hand, the visual field restriction for people with peripheral vision loss (PVL), such as those with retinitis pigmentosa (RP), greatly affects their visual search ability despite having good visual acuity [14], [15] They may not have problems in discerning the scene details, but often have difficulty in knowing where to look for the required information and may need help in locating the targets (Figure 1b). Thus, visual search related challenges could arise due to a variety of reasons, including inability to locate the targets, inability to discern target details, or both. Therefore, a vision aid assisting in visual search can help people with vision loss in various daily life tasks.

To tackle the visual search related challenges in visually impaired users, dedicated devices [16] and services [17] have been developed that leverage artificial or human intelligence. Considering the growing prevalence of mobile devices in the general as well as visually impaired population [18]–[21], mobile applications aiding in visual search specifically targeting visually impaired users are also now available. [22], [23] The main idea behind many of these vision aids is to perform one or multiple functions including object detection, optical character recognition (OCR), and/or scene categorization using computer vision, and then provide some feedback to the user via a predefined tags or descriptions. Performing generic object detection can be challenging in the real world (for example, product identification based on barcode or appearance) and the predefined categories of object classes may be too restrictive to cover the rich variety of objects encountered in everyday situations. Comparatively, OCR is a much more well-defined problem with mature and established technologies available to tackle it. Searching with keywords can be intuitive and help narrow down the scope of the search, thereby improving the odds of obtaining the required information. However, dedicated OCR apps are meant for document reading instead of detection of text in the scene [24] Even in applications that can perform OCR in scene images, the feedback provided to the user is generally not relevant, as the entire text blocks detected in the scene are continuously read to the user. We have developed a mobile app, Supervision Search (SVS) [25], that can perform keyword search in scene images so that the users can quickly and efficiently retrieve the relevant information from their surroundings or from the object of interest.

The key arguments in support of development of SVS app are: i) searching for items represented by text or symbols forms a large part of visual search activities in the daily lives of people with low vision, ii) in most cases, people already know what they are searching for (i.e., the keywords are already known), and iii) if the keywords are located in the scene, then the information related to those keywords is available in the general vicinity (spatially) of the detected keywords. Searching with keywords, for instance using Google, is ubiquitous when the information is in digital form. The SVS app merely generalizes the same approach in real-world scenes. By leveraging powerful OCR engines, the SVS app searches for user input keywords, then highlights and zooms in on the found instances of the keyword in the captured scene image to facilitate quick and easy retrieval of information related to the keyword.

In this paper, we describe the concept of keyword search in natural images and its relevance in various daily life activities, detail the design of the SVS app to target different requirements of low vision users, and present results of its preliminary evaluation: both of the underlying algorithms, and of the app by human subjects with simulated visual acuity loss. The goal of this work is to test whether the approach of keyword search in natural scene images can be utilized to design a vision aid for visually impaired people, and whether the SVS app can provide additional benefits compared to conventional visual aids.

## II. Keyword Search in Scene Images

For developing a visual search assistance application for visually impaired people, we needed to identify methods for performing the search, as well as define methods for seamless interaction with the user. For performing the keyword search, we relied on established optical character recognition (OCR) technologies instead of developing custom algorithms from scratch.

### A. Optical Character Recognition Engine

An OCR engine lies at the heart of a keyword search application, as its capabilities and limitations essentially shape much of the usability of the search application. Quite simply, an OCR engine processes the input image to detect text regions, recognize the characters, group them into words, and output strings of text with associated metadata, such as its location in the image (coordinates of the bounding box for a word), and possibly its orientation with respect to predefined axis, among other data. The main application for OCR technology has been to digitize printed documents; and in the space of assistive technologies for visually impaired people, OCR is extensively used in applications for assistance in reading printed material and documents [24] However to be truly useful in general scenarios, OCR needs to work for text embedded anywhere in the scene, and not just documents. Thus, scene text recognition is more challenging and cannot be handled very well by applications focused on document OCR processing. Therefore more sophisticated OCR engines are needed.

Fortunately, there are various commercially available $3^{\mathrm {rd}}$ party OCR engines, available for incorporation within end-user applications, which have been trained using advanced machine learning techniques to work in highly demanding real-world images containing text. Four different OCR Engines that can be implemented via an API were evaluated to determine the accuracy and usability of each engine: Google Machine Learning Kit (ML Kit) [26], Microsoft Azure’s Cognitive Services (Azure OCR) [27], ABBYY’s Real Time Recognition SDK (ABBY RTR) [28], and Amazon’s Rekognition SDK (Rekognition) [29].

• Google Machine Learning Kit (version 16.0.0) — This service provides machine learning models for text recognition that are both native to the device and cloud-based. In this study however, we only used the native version of the API that is freely available. This engine returns the words that are found in the image along with the coordinates of the bounding box of each word.
• Microsoft Azure’s Cognitive Services (version 2.0) — This cloud-based service provides image processing algorithms to identify content present in the image. Pictures of the content are sent to the service to be processed, and a JSON file is returned with the detected text, a bounding box for each word, and its orientation angle. There is a limitation on the maximum image size (4MB) that can be sent to the service. Being a cloud-based engine exclusively, it requires a network connection to work and is not particularly well-suited for real-time applications.
• ABBYY’s Real Time Recognition SDK (version 1.0.7.56 free demo version) — A native model similar to Google’s Machine Learning Kit, this OCR Engine can also process frames of a live video stream in real time. A confidence level for detection needs to be set as an operating parameter for accuracy of detection of a word within the image. This engine returns the location of the line in which the word is present within the image, but the exact bounding box for a particular word was not available for the version used in this work.
• Amazon’s Rekognition SDK (version 2.6) — This is a cloud-based service that returns the detected text, the confidence level of each word, and the “geometry” of the word, which contains a polygon that surrounds the word. A limitation of this OCR service (the version that was available for this work) was that the image size sent to the cloud server was restricted to 5MB, and it only processed up to a maximum of 50 words within the image.

### B. Evaluation of OCR Engines

**FIGURE 2.**
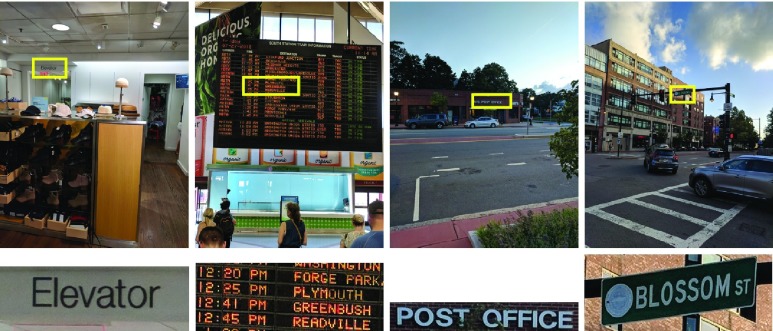
Examples of natural images (captured with a mobile device) used for testing the OCR engines. Actual images used for testing are shown the in the top row and the search keyword associated with each image is listed in the bottom row. The location of the keyword in the images is highlighted in the images with an overlaid yellow rectangle (for the purpose of illustration, does not reflect OCR output). The highlighted patch is zoomed in the middle row below the images. The keywords for each image were chosen to represent realistic usage in way finding scenarios.

The accuracy and robustness of the above 4 OCR engines in finding a specific search query was evaluated in a variety of natural images captured in offices, train stations, stores, and on streets in downtown Boston (Figure 2). For each image, a set of most relevant keywords were identified to be tested by the OCR engines. An image could have more than one keywords associated with it. Keywords were selected such that they would make sense in a realistic application. For example, in an image of an intersection, the likely search query would be the street name present in the image; or for a food product, the likely search queries could be any of the nutritional factors. A total of 203 keywords for 117 images were identified. High resolution images were captured using Samsung Galaxy S7, S8, and Google Pixel smartphones; although the images were not of exact same size (Mean ± SD diagonal was 4939 ± 258 pixels). More specifically, the 117 test images captured from the three smartphone devices had one of these 4 resolution values: $1520\times 2688$ (n=2), $2268\times 4032$ (n=31), $3024\times 4032$ (n=12), and $3036\times 4048$ (n=72). Each image was tested at 3 different zoom levels — $1\times $, $2\times $, and $4\times $. If an OCR engine was not able to find the keyword at the lowest zoom level, then next zoom level was tested. Images were zoomed such that the search keyword was present near the center of the image. The Lanzcos interpolation was implemented, as it allows for detailed upsampling of the image and preservation of small text. The true location of the keyword within the captured images was determined manually for comparison of the spatial accuracy of the keyword localization. For each keyword, the detected text and the location of each word was saved (with the exception of ABBYY Real Time Recognition service, where it was not possible to obtain the location of the detected keyword from the SDK version that we were using for testing).

The performance of the OCR engines was evaluated in terms of the number of keywords successfully found, and the zoom level at which they were found. Particularly, we were interested in determining the cumulative detection success and the success in detection at the base scale (no zoom or $1\times$ condition) for performance considerations. For both of these outcomes, the proportions of successfully detected keywords were compared between the 4 OCR engines. The closeness of the location of the detected keyword in the image to the ground truth location was also determined. The distance (Euclidean) between the detected word and the ground truth location was calculated and normalized with respect to the image dimensions. For determining the localization accuracy, only successfully detected single keyword instances were considered. Log-transformed keyword distances were compared between the 3 OCR engines, excluding ABBYY RTR for which we did not have data.

### C. Results

**FIGURE 3.**
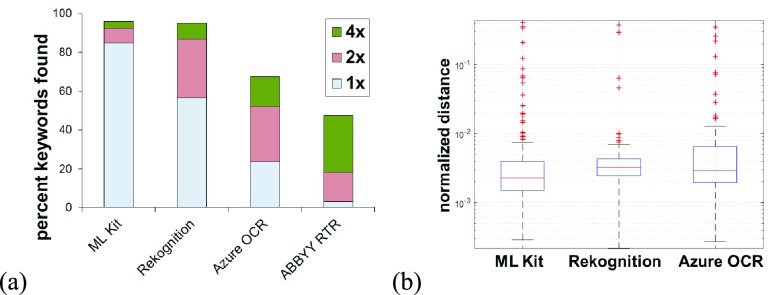
OCR testing results in natural images captured by a mobile device. (a) Comparison of the percentage of keywords successfully found by the 4 OCR engines tested in our study (from a total of 203 keywords over 117 images) at different image zoom levels. Cumulative percentage of detected keywords for ML Kit and Rekognition were significantly higher than others. ML Kit was also significantly better than the other 3 in successful detection at base zoom level ($1\times$). (b) Comparison of the average normalized distance to the detected keyword in the image from the ground truth location (manually marked for each keyword). Keyword localization error was significantly different between Rekognition and ML Kit.

Out of the 203 keywords tested, only 3 were not detected by any of the OCR engines. A 4-sample test for equality of proportions showed that the overall successful search rate differed significantly between the OCR engines ($\chi ^{2} =187.5$, df = 3, p < 0.001), with ML Kit and Rekognition having a significantly higher overall success rate than Azure OCR and ABBYY RTR (p < 0.001 for all multiple pairwise comparison with Bonferroni correction). The proportion of successfully detected keywords was significantly higher for Azure OCR compared to ABBY RTR (p < 0.001). There was no difference in the overall success rate between ML Kit and Rekognition. Successful search rate at $1\times$ zoom level differed significantly between the 4 OCR engines ($\chi ^{2} =325.16$, df = 3, p < 0.001), with ML Kit being the most successful in detecting keywords without needing to zoom in, followed by Rekognition, Azure OCR, and ABBYY RTR. All pairwise comparisons were significant (Bonferonni correction). While the overall successful detection rate was not significantly different between ML Kit and Rekognition, ML Kit detected significantly more keywords at the base zoom level (Figure 3a). The keyword search success rates at different image zoom levels for the 4 OCR engines are shown in Figure 3a and summarized in Table I. The keyword detection accuracy did not change significantly between different image resolutions, when blocking for scale at which they were found (Friedman’s test: $\chi ^{2}=3.9$, df = 3, p = 0.27).

**TABLE 1:** Successful Keyword Search Rates at Different Zoom Levels for the 4 OCR Engines

	ML Kit	Rekognition	Azure OCR	ABBYY RTR
$1\times$	172 (84.7%)	115(56.6%)	48 (23.6%)	6 (3.0%)
$2\times$	16 (7.9%)	61 (30%)	58 (28.6%)	31 (15.3%)
$4\times$	7 (3.4%)	17 (8.4%)	31 (15.3%)	59 (29.1%)
Total	195 (96.1%)	193 (95.1%)	137 (67.5%)	96 (47.3%)

**FIGURE 4.**
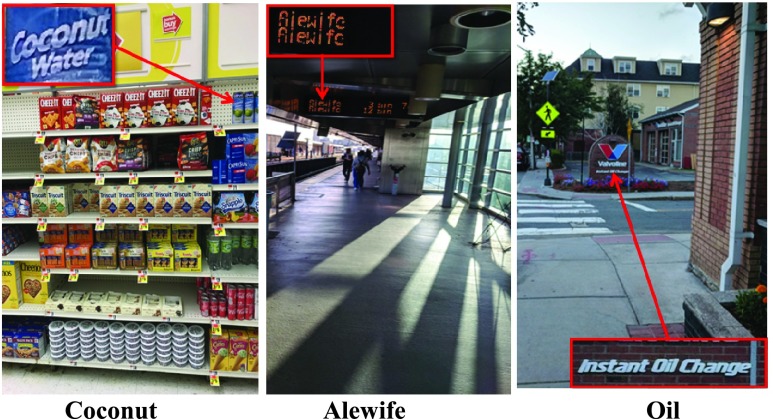
Some examples where the OCR engines failed to detect the keywords. The keywords associated with the images are shown below the pictures. The location of each keyword within an image is indicated by a red arrow, and the keyword region is shown in the inset for clarity. Font type variations, background clutter, and image degradation are some of the main reasons for OCR failure in natural images.

The keyword localization accuracy was evaluated by computing the distances between the keywords correctly found in the image and the ground truth locations, based on 164, 163, and 113 cases out of 203 for ML Kit, Rekognition, and Azure OCR, respectively. The keyword localization errors were significantly different when comparing the three engines (Kruskal-Wallis $\chi ^{2} =10.54$, df = 2, p = 0.005), with pairwise comparisons revealing a significantly larger localization error for ML Kit compared to Rekognition (p = 0.003 with Bonferroni correction). There was no significant difference between Rekognition and Azure OCR. Relative to the size of the images, the keyword localization errors were between 0.2% and 0.4% of the image dimensions. Some examples of keyword search failures are shown in Figure 4.

### D. Discussion

Our evaluation of the OCR engines with natural images shows that ML Kit performs the best in terms of proportion of keywords successfully recognized, as well as the proportion of keywords recognized at the base image scale without needing to zoom in. Not having to zoom-in for successful recognition can potentially save processing time and maintain the maximum possible the field of view of the scene, thereby improving the usability of the search paradigm in complex real world scenarios. Even though ML Kit was found to be statistically significantly worse at localization of keywords but overall just slightly (localization error of 1.4% of the average image size) compared to Rekognition(error of 1% of the average image size), the differences are mainly due to outliers. One of the main reasons for this is OCR mistakes, such as the merging of the keyword with neighboring word (for example, when searching for keyword ‘pharmacy’, the localization is affected by the preceding text ‘CVS’. So while the keyword is found, the localization error with respect to the ground truth location can be higher. While highly precise localization of the detected keyword in the image is an important consideration, a small localization error can be tolerated when searching for information associated with the keyword. As previously reported, the 0.2 to 0.4% localization error amounts to less than 20 pixel difference, which is negligible considering the information associated with the keyword will still be in the neighborhood of the searched keyword.

One of the main reasons for search failure, particularly for OCR engines other than the ML Kit, was the small size of the text in the image, which was resolved in most cases by increasing the scale. Other reasons were image degradation and presence of non-standard fonts (such as too artistic, oriented in different directions, or composed of dots as seen in the LED display on train station in Figure 4). Another factor that could be responsible for search failure is OCR errors, where the recognized text from the image contains the keyword but OCR mistake such as merging, typos, or special characters prevents its successful identification.

There was a large disparity in the capabilities of the OCR engines to detect slanted text. ML Kit and ABBYY RTR were limited to detection of text in a relatively narrow range of orientations (≈±30°). Azure OCR was able to detect text in all possible orientations due to its ability to switch the axes of the images based on the device orientation. However, it assumed that all the text within the image had similar orientations and thus could not handle multiple orientations of text within the same search image. Rekognition was able to handle text in multiple orientations.

From the usage-standpoint, each OCR engine had its own strengths and limitations. Rekognition and Azure OCR were cloud-based engines and thus required network connection. Given the lag in transmitting the input image and retrieving the detected text within the image from the cloud, real-time implementation with these OCR engine is not practically feasible. Moreover, the version of Rekognition SDK tested in this study had an upper limit on the number of words it could detect in a given image. Thus, despite being relatively successful in detecting almost all the keywords in our outdoor test images, it could not handle situations where there was a lot of text in the image. The ABBY RTR engine could be implemented natively (not requiring cloud-based processing), but the demo version we tried in this study was clearly inferior to other OCR engines in scene text detection. Comparatively, ML Kit worked natively on the device (did not require cloud-based processing), was capable of providing real-time text detection output, and showed robust performance in challenging images, particularly detecting text without needing to zoom-in. Based on the experimentation with the OCR engines, we chose ML Kit for implementing the Android version of the SVS app with the capability for real-time scene text detection.

It should be noted that the OCR engines in our experiment could only be accessed via standard API calls, and therefore were essentially treated as black-boxes. It is possible that there were internal details no known to us that led to their differing performance in our experiment. For example, it is possible that MLKit OCR was using some kind of multi-scale approach internally that made it more successful in detecting keywords at base image scale. However, our comparison treats all the OCR engines on the same level, i.e., using them in an “as is” state based on the available specifications for the application developer.

## III. The Supervision Search (SVS) Mobile Application

### A. Description of SVS App

**FIGURE 5.**
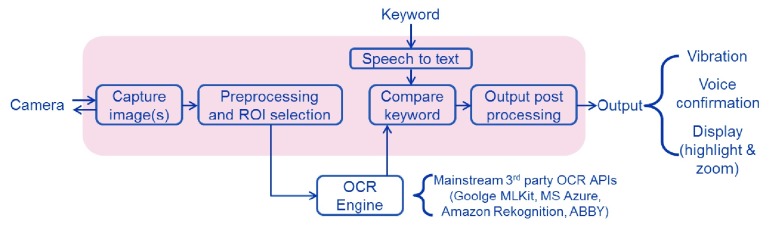
Overview of the operational steps in performing keyword search in natural images using a mobile device. The shaded region represents the block diagram of the Supervision Search app.

The operational concept of the SVS app is shown in Figure 5. The scene is captured by the device’s camera and the image(s) are processed by the OCR engine to detect all the text present in the image(s). The keyword input by the user is searched for within the detected text, and depending on the usage mode, the user is informed via a combination of various methods: vibration of the device, voice confirmation, and displaying the zoomed-in and highlighted instance of the keyword. The keyword can be input via speech (standard voice input in the mobile device) or via keypad. The underlying OCR engine can be implemented using any of the mainstream APIs, including the 4 OCR engines discussed above. The application interface remains the same irrespective of the choice of the OCR engine, although the choice of OCR engine affects the performance and other operational parameters (such as availability of a network if the OCR API is cloud-based). The implementation of the app is constrained by the off-the-shelf OCR API itself. The OCR engines typically take the entire image as the input and provide all the scene text detected in the input image as the output. Therefore, keyword comparison has to be performed following the OCR stage. Currently, the iOS version of the SVS app is based on Azure OCR. The Android version of the SVS app has been implemented using the ML Kit due to its native processing, real-time performance, and its overall robustness. The app functionality explained below pertains to the Android version.

**FIGURE 6.**
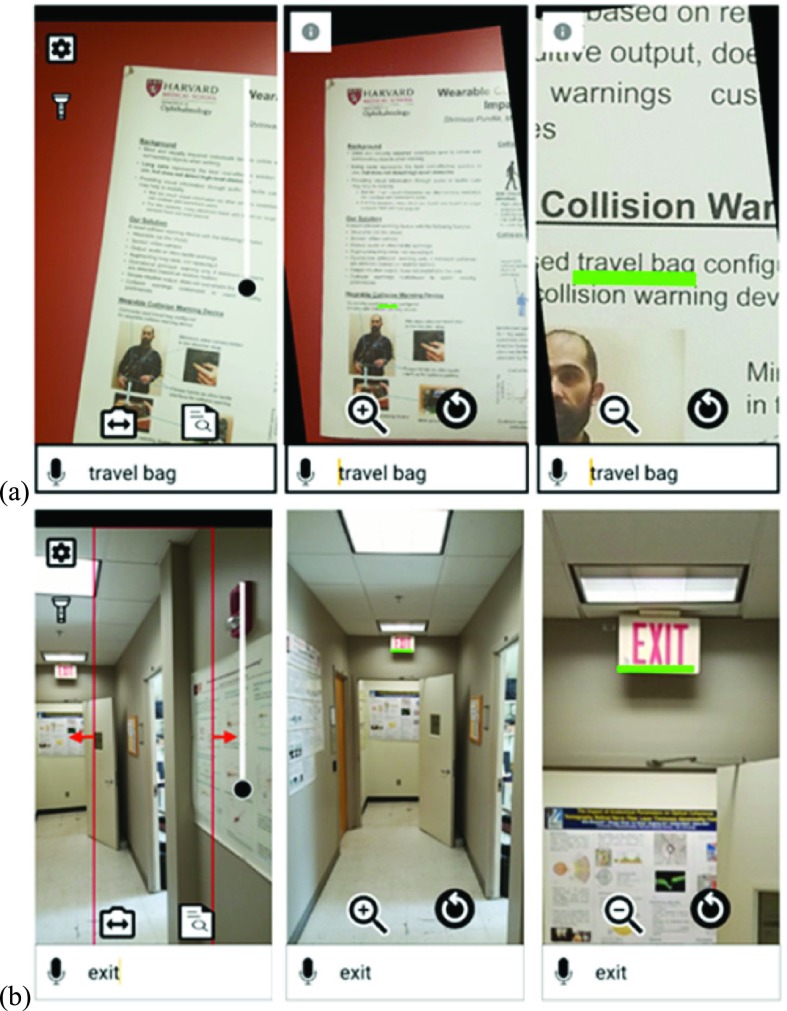
The two operational modes of the SVS app. Screenshots of the input screen, the search notification screen, and the zoomed in location of the found keyword are shown for each mode. (a) In the point-and-shoot mode, the user takes a picture of a scene and the search result is highlighted and zoomed in for better viewing. (b) In the real-time scan mode, there is a region of interest at the middle of the screen. When the keyword is found within this region of interest, then the app provides an indication. Thus the direction of camera pointing is loosely related to the spatial location of the keyword in the scene. This helps in way finding, for example- in this case, the location of the exit sign.

There are two modes of operation for the SVS app: point-and-shoot mode and real-time scan mode (Figure 6). The point-and-shoot mode is ideal for searching a keyword in the scene or within an object. On the other hand, the real-time scan mode is more suited for searching in a wide field of view, for example: localizing the exit door. In point-and-shoot mode, a single high resolution picture of the scene is captured with the rear camera of the mobile device and processed by the OCR engine to detect the presence of the keyword input by the user. In the real-time scan mode, the user presses and holds on the screen while moving the device in a scanning pattern over the target area (for example, scanning with the mobile device from side to side). Each video frame acquired by the camera is grabbed from the image buffer and processed by the OCR engine to detect the presence of the keyword in the scene in real-time. With the real-time search, when the keyword is detected in the scene, the orientation of the device with respect to the user will approximately indicate the direction of the found keyword. In order to improve the localization precision of the keyword in the real-world, a scanning slice is introduced to restrict the field of view of the camera, thereby reducing the search area (searching only within a narrow strip of each frame’s image). This scanning slice is a user selectable option and is set as the central 50 percent region of the screen (horizontally) by default for real-time scan mode. By reducing the region of interest for search, the physical location where the device is pointing aligns more closely to the actual target location, enabling users to orient themselves more accurately with the detected target and reduce the negative impact of motion blur from the movement of the device when scanning.

**FIGURE 7.**
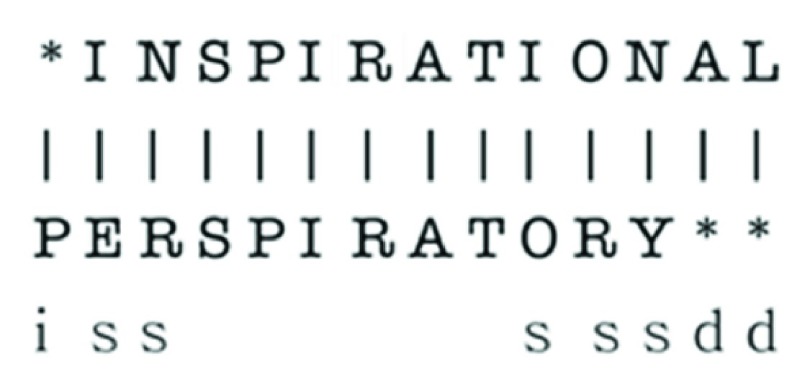
An example showing how the modified levenshtein distance is calculated when comparing two words. In this graphic, ‘i’ represents an insertion, ‘d’ represents a deletion and s represents a substitution. Punctuation and plural words are ignored in the algorithm. Hyphenated words are regarded as multiple separated words. The distance between inspirational and perspiratory is 8. Due to fact that perspiratory is a shorter word – with 12 characters – the modified algorithm would return a value of 0.67. This number is greater than the tolerance value in the app and thus these words would not be considered a match.

To minimize the impact of inevitable errors in OCR and possible typos in provided keywords, the SVS app does not require an exact match to keywords. Instead, the matching is based on a modified Levenshtein Distance [30] When comparing the keyword with each word in the detected text, this algorithm determines the number of insertions, deletions, and substitutions that are required to change the detected word to the search keyword (Figure 7). Thus, with more edits, the probability that the candidate word is the search query diminishes. The net number of edits in each word is then divided by the length of the smaller of two words being compared to generate a distance value. This normalization allows the comparison algorithm to be dynamic — allowing for more edits in the detected word with a longer search query. Finally, the distance of each word in the detected text with the keyword is compared with a threshold. If the distance is lower than the tolerance, then the given word in the detected text is considered a match to the input keyword. This threshold value was set at 0.3 for this study. Such an approach for string comparison is useful in dealing with OCR inaccuracies, as well as tolerating minor differences in the input keyword and the actually present word (such as handling plurals or other minor variations or typos made by the speech to text).

When the searched keyword is found, it is highlighted in the image by a flashing green bar to make it more visible to the user. At the same time, voice output from the device indicates the number of instances of the keyword that were found in the image. In addition, the app allows the user to directly zoom in on the highlighted keyword to retrieve any necessary information related to it that is present in its immediate neighborhood. In the case of multiple instances of the keyword being present, the app allows the user to zoom in on each instance sequentially. The initial zoom level is adjusted based on the size of the detected keyword relative to the display, so that maximum zoom is applied while keeping the entire keyword in the screen. Thereafter, the user can change the zoom as needed using a pinch gesture. The user also has the option to search for another keyword within the captured image. The highlighting and zoom parameters are updated accordingly. In the real-time scan mode, the app indicates the successful detection of the keyword in the image (in real-time) by vibration. Thus, as the user is scanning with the mobile device, instant feedback is received when the keyword is found. This helps in associating the presence of the keyword within the captured image with its actual location in the scene via proprioception.

Handling of the rotated text for display purposes has to be done outside of the OCR engine. While the native ML Kit OCR engine is restricted to detecting text with an orientation of ±30° with respect to the horizontal direction, the exact orientation of the detected text is not returned by this engine. Thus to display the rotated text (unidirectional rotation only) properly, the rotation needs to be corrected for. This is done by computing the average orientation of the entire text from the bounding box information for each detected word and then cancelling the amount of rotation with this average orientation when displaying or highlighting.

### B. Evaluation of the SVS App

Preliminary evaluation of the point-and-shoot mode of the SVS app was conducted with human subjects to determine whether the concept of keyword search can provide any benefit in performing visual search activities in daily life. We recruited 6 adults with normal vision (best corrected visual acuity 20/20 or better and no other known vision disorders) from our institute for performing the app evaluation. During the experiment, they wore blur glasses (blurring filter attached to a no-power lens) that reduced their visual acuity to the level of 20/100 – 20/125. The study followed the tenets of the Declaration of Helsinki and informed consent was obtained from all the study participants. The protocol was approved by the institutional review board at the Massachusetts Eye and Ear Infirmary.

The evaluation task was a variation of the timed instrumental activities of daily living (TIADL) task [31], [32] The TIADL tasks consist of a sample of visual activities routinely performed in daily life such as reading ingredients of food products, instructions on medicines, finding phone number in a directory, and using tools, among others. In our study, we curated tasks that required keyword search, increased the overall number of tasks to be performed, and changed the complexities of the tasks such that they varied from simple to complex (difficult). While task difficulty is a relative term, in this study difficulty refers to the time required to complete the search (find the keyword).

Briefly, these included finding specific information from the items such as finding due date on a utility bill, or whether the food product contained nuts, or finding lowest priced clothing item from a catalogue. The items were classified into 5 categories: printed sheets (flyers, utility bills etc.), documents (booklets, brochures etc.), restaurant menus, food products, and other household items (Table II). Overall, 50 items were selected and split evenly in two conditions where the subjects searched for the information either using the SVS app or using handheld optical magnifiers ($4\times $ and $12\times $). Pilot testing was done to make the overall grouping of the items balanced in the two conditions (items in both groups were at about same difficulty level).

**TABLE 2:** Modified TIADL Tasks Used for Evaluation Study. A Total of 50 Items Were Identified and Were Divided Equally in Two Sets to be Tested in Two Conditions: While Using the SVS App and While Using Optical Magnifiers

Item Category	Description	Typical Information Requested	Number
Printed sheets	• Utility bills • Bank Statements • Flyers • Forms (Tax etc.)	Due date, amount due, amount related to a particular transactions, reward balance, contact info on the flyer	15
Documents	• Telephone directory • Brochures • Catalogues • Manuals	Telephone number of a particular business or a person, price of an item in the catalogue, specifications listed in the manual	12
Restaurant Menus		Price of items containing a specific kind of food (for example, chicken, salmon etc.)	6
Food products		Nutritional information, ingredients	5

The search task administration to the subjects occurred by presenting an item (each trial consisted of a different item) and asking them for specific information contained within the item. The task question was framed in a manner such that the intended search keyword was either part of the question or was implicit in the question. However, they keyword was not directly provided to the subjects, and they were supposed to come up with the keyword on their own when searching for the requested information. For example, if the keyword to be searched in a credit card statement was reward points, the subjects were asked to report what was the reward point balance for the month. The examiner would time the task (using stopwatch) after presenting the item and communicating the keyword for it. The timing stopped when the subject responded with the correct answer (the trial continued if the answer was incorrect for any reason). For the SVS app condition, timing of the trial included keyword input time and the search time with any subsequent errors due to inputting an incorrect keyword, speech recognition errors by the voice input functionality of the smartphone, OCR related errors, or any other user errors. When using optical magnifiers, the subjects were free to choose from $4\times $ and $12\times $ magnifiers depending upon the text size in the item. SVS app was run on a Samsung Galaxy S8 smartphone. Subjects were trained to use the SVS app and optical magnifier for searching with some practice items before the actual trials. The order of device use and task group was counter-balanced. The experiment was conducted in a well lit room (standard office room lighting). The total study time for a subject was about 2.5 hours including task training (≈30 minutes) and a break of ≈10 minutes between the two conditions.

An upper limit was placed on the search time for each trial (360 seconds) and if the subject did not find the required information within this time the trial was ended and the search time for that trial was set as 360 s. For determining the benefit of the app in visual search, we compared the cumulative task time between the two conditions. Cumulative task time was the sum of search times for individual trials in a given condition. Since the individual tasks varied a lot in terms of their difficulty within the conditions, we also performed further fine grained comparisons by considering task difficulties. The search times for a subject were sorted from fastest to slowest for each condition. The average sorted task time was compared between the two conditions. In the sorted task list, a crossover point was determined for each subject after which the tasks were increasingly difficult to perform manually compared to the app. Tasks sitting at a higher order in the list compared to the crossover point were therefore considered difficult to perform manually using the magnifier. Comparisons of the median task time before and after crossover point were done between the two conditions.

### C. Results

**FIGURE 8.**
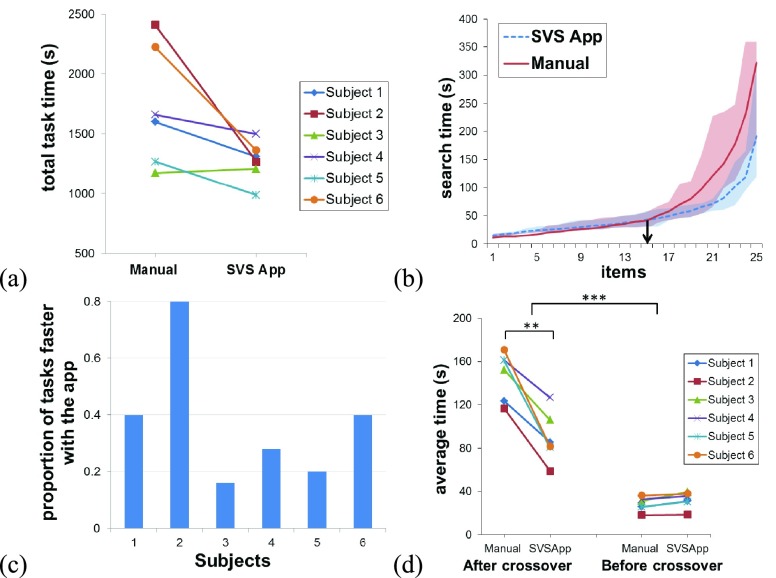
Evaluation of SVS app with normally sighted subjects wearing blur glasses (n=6). (a) The cumulative task time (sum of individual trial times) reduced when using the SVS app in all but one subject (#3). In some subjects (for example #2 and #6), there was a drastic reduction compared to manual search with a magnifier. (b) Average sorted search time across subjects with and without the SVS app. The shaded region shows the limits (max. & min.) of the response for the respective conditions. The plot shows that the search tasks were of variable difficulty levels ranging from simple to difficult. Difficult tasks took longer to complete. The crossover point (denoted by downward pointing arrow on the chart) can be determined from these curves: the point after which search with the optical magnifier takes longer compared to the SVS app. (c) Chart showing the proportion of the tasks that were faster with the SVS app for each subject. These correspond to the tasks that sit at a higher order than the crossover point for the given subject. (d) Average task time before (easy) and after (difficult) the crossover point between the two conditions. For the difficult tasks, the median search time with the app decreased significantly. However, there was no statistically significant difference in search time between the two apparatuses for easier tasks. ** denote p < 0.01, *** denote p < 0.001.

Cumulative task completion times between SVS app and optical magnifier were not significantly different, but the difference approached significance level of 0.95 (paired t-test: t = 2.4, df = 5, p = 0.059) (Figure 8a). The cumulative task completion time reduced with the app for 5 out of 6 subjects (average ± std. for 6 subjects: manual method = 29 ± 8 minutes, with app = 21 ± 3 minutes). For the one remaining subject, cumulative task time with the app was only about half a minute more than manual searching. However, the cumulative task time does not fully inform us about the underlying data given the task variety and the variable difficulty level of the tasks, which is evident with the skewed average sorted search times (Figure 8b). It can be seen that search with magnifier was almost as fast as the SVS app for the items before the crossover point for the two curves (easier tasks). However, over the remaining items beyond the crossover point, the SVS app appeared to be substantially faster. The crossover point varied between subjects (Figure 8c), indicating that the relative proportion of the search tasks for which the app was faster compared to manual search varied between subjects. Averaged over 6 subjects, about 37% tasks were faster with the app.

We then compared the average search times for tasks before and after crossover point for each subject with and without the app using repeated measures ANOVA. There were significant effects of the search method (F(1,5) = 30.5, p = 0.003), crossover point (F(1,5) = 231.4, p < 0.001), and their interaction (F(1,5) = 47.8, p < 0.001) on the average search time. Post hoc tests with Bonferroni correction showed that searching with app was significantly faster than manual searching for the more difficult tasks (tasks beyond the crossover point) (average of 6 subjects - without app: 148 ± 22 seconds; with app: 90 ± 23 seconds, p = 0.009). The difference in the average search times between the two methods before the crossover point (easier tasks) was not statistically significant (without app: 28 ± 6 seconds; with app: 32 ± 8 seconds, p = 0.12) (Figure 8d).

Over 150 trials with the app across 6 subjects (25 each), there were 12 app errors such as OCR failures (8%), 16 keyword input errors (10.7%), and 9 user errors such as incorrect app operation (6%). On average (± std.) each subject encountered 2± 1.55 app errors, 2.67± 1.21 keyword related errors, and 1.5± 0.55 user errors. There errors were not necessarily on different tasks, some tasks encountered multiple kinds of errors. When searching with the magnifier, subjects used $4\times $ magnifier for 78% of the tasks, $12\times $ magnifier for 8% of the tasks, and both for 14% of the tasks.

### D. Discussion

The evaluation results show that SVS app can lead to an overall saving in the search time, particularly for complex search tasks that are deemed to be difficult. On an average, there were savings of ≈7.5 minutes (26% reduction) with the app compared to search with the optical magnifier for cumulative task time. But the cumulative search time does not tell the entire story since the specific tasks used in this study were somewhat arbitrary. As Figure 8b indicates, the search times increase exponentially as the tasks get more complex. This is true for both with and without app conditions. It can be expected that including more difficult tasks would lead to more time saving, or vice versa. Therefore, the absolute time saving in cumulative search time with the app is not very meaningful in the sense of generalization. Previous studies using the TIADL methodology tested with a limited number of tasks, for instance 17 in Owsley *et al.* [31], 5 in Taylor *et al.* [33], and 3 in Wittich *et al.* [34]. It is arguable that too few tasks may not be able to capture the variety of situations encountered by people in their daily living.

In this evaluation study, we included 50 tasks with a wide range of difficulties, in an attempt to generalize the study findings. A key consideration is not simply the number of tasks, but the difficulty range. In Figure 8b, we can see that the search time with magnifier appeared to increase more rapidly compared to the SVS app, as the difficulty level of the tasks increased. This finding will remain unchanged, i.e. it is generalizable, no matter what tasks are included, as long as the range of task difficulty is sufficiently broad. To perform more detailed analysis, we employed the method of separating the tasks around the point where the sorted search time curves for the two apparatuses cross over (the crossover point), essentially separating search tasks into two categories: easy vs. difficult. Separating the tasks around the crossover point allowed us to understand the benefit of the app across broad range of task difficulty as well as for different individuals. The results clearly indicate that for the easy tasks the search time was almost the same for both the apparatuses, but for the difficult tasks the SVS app helped in reducing the task time by a large margin (close to 40% reduction as compared to the optical magnifier).

The search time reduction with the SVS app is not seen across the board for all items because the app requires some fixed amount of setup time, including inputting the search keyword and taking the picture. Thus, the SVS app may not be time-saving when the tasks are easy (for example, when searching in less cluttered objects). It should be noted that the search time for the SVS were inclusive of instances of failure of keyword detection due to the following reasons: subjects inputting a keyword that was not present in the item (or inputting an incorrect keyword), failure of OCR engine due to image capture issues (such as blur or specular reflections off of the surface of the objects), failure of search due to incorrect pointing of the camera (keyword out of the field of view of the camera), and incorrect speech recognition (voice input returning incorrect keyword). On the contrary, searching with the optical magnifier did not require any overhead time: the subjects could start the search the moment they heard the question for the given task. The SVS app was able to reduce the overall search time in complex tasks despite these limitations, errors, and inaccuracies.

The human subject evaluation was currently limited to the point-and-shoot mode of the SVS app. The ability of the OCR engines to successfully search in images of outdoor scenes, as shown in this paper, lends a lot of promise to the utility of real-time scan mode. Future work should evaluate the real-time scan mode for the purpose of way finding. The human subject study sample was small and was also limited to normally sighted individuals with simulated visual acuity loss. Therefore, they were not habitual users of optical magnifiers or habituated with searching with reduced visual acuity. Also, there was not much variation in the visual acuity levels of the study subjects. These reasons somewhat limit the validity and generalizability of this study. However, the longer search times in manual search condition indicate that loss of visual acuity was a factor in search performance. The real benefit of the app can only be determined after performing a study with visually impaired people, which will be future work. In future, we will also include subjective opinions of the low vision participants regarding the app and their preference relative to using habitual search aids.

## IV. General Discussion & Conclusions

In this work we have shown that keyword search in real-world scenes can be implemented in a vision-assistive mobile application, and it can help make keyword-based searches faster and easier in real-world visual search tasks when visual acuity is lowered. Our specific keyword search offers a direct and realistic chance of returning a successful hit, and consequently providing the users with the related information without overloading them with irrelevant information. Use of the mobile platform is justified due to the increasing popularity of mobile devices, even in the elderly population [18], [21] Furthermore, vision aids can be made cheaper and more accessible if delivered via mobile platform. In the support of implementing keyword-based vision search as a vision aid, there is evidence that spot reading is highly prevalent in people using mobile app-based smart vision aids. [35] Spot reading is required for quick information gathering tasks and thus, a keyword searching application can help in spot reading and information acquisition.

One limitation of this approach is that the user needs to input a keyword that is present in the scene (or a close variation of it), which may not always be the case. This was observed in our human subject testing of the SVS app, where the subjects entered keyword that was not present in the scene and thus they had to retry with a different keyword. We did not explicitly provide the subjects with the keywords they were supposed to search, and instead expected the subjects to come up with their own relevant keyword for the given task. This study design was more realistic in simulating the daily life tasks where subjects are generally aware of likely keywords but do not explicitly know which keyword they should search for. Although we have designed some tolerance for typos introduced by the OCR engines or minor changes in the keyword depending on the context (for example, plurals ending with an ‘s’), our approach cannot deal with synonyms of a keyword. In the future, we will deal with the issue by introducing a smarter search that can suggest context-based synonyms of the input keyword if a direct match is not found.

In conclusion, this work describes a novel vision aid implemented on a mobile device that can help make visual search related daily life tasks easier to perform by visually impaired people. Impaired vision is a complex condition and loss of visual acuity is only one aspect of it. Still, the preliminary results shown in this paper support the approach of keyword searches in real-world scenes for aiding visual search. The SVS app can be potentially beneficial to the people with low vision in complex search tasks, though further investigation is required. In future studies, we will evaluate the benefit of the app in visually impaired users.
